# Volatile Infochemicals from *Rhyzopertha dominica* Larvae and Larval Feces Involved in *Theocolax elegans* Host Habitat Location

**DOI:** 10.3390/insects12020142

**Published:** 2021-02-07

**Authors:** Giulia Giunti, Orlando Campolo, Pasquale Caccamo, Francesca Laudani, Vincenzo Palmeri

**Affiliations:** Department of Agriculture, University Mediterranea of Reggio Calabria, Loc. Feo di Vito, 89122 Reggio Calabria (RC), Italy; pako.caccamo.97@gmail.com (P.C.); francesca.laudani@unirc.it (F.L.); vpalmeri@unirc.it (V.P.)

**Keywords:** behavior, biocontrol, Bostrychidae, integrated pest management, parasitoid, Pteromalidae, stored product

## Abstract

**Simple Summary:**

Food protection is a key issue to guarantee food security. One of the major criticisms is related to insect pests, which can severely damage stored products. Control of stored product pests widely relies on synthetic pesticides, which are potentially harmful to human health and the environment. In this context, the application of chemicals during post-harvest should be limited, and natural enemies, like parasitoid wasps, might be useful for biologically based pest management programs. The effectiveness of this approach under field conditions is still uncertain, and more information about parasitoid biology and behavior can be valuable to improve mass rearing and control strategies. This study investigates the host habitat location behavior of *Theocolax elegans*, a generalist parasitoid attacking several stored product pests, including *Rhyzopertha dominica*, a key pest species of stored grains. Bioassays demonstrated that the parasitoid females were not attracted by intact kernels; indeed, the parasitoid females were strongly attracted by infested wheat and by the host feces, locating the suitable hosts through the characteristic volatile infochemicals emitted by these substrates. Results from the present research are encouraging and suggest that biological control agents might be used to reduce the amount of synthetic insecticides.

**Abstract:**

The development of biologically based approaches for stored product pest control is needed to reduce chemical inputs. Bioassays were performed to investigate host habitat location in the trophic interaction durum wheat/*Rhyzopertha dominica*/*Theocolax elegans*. GC-MS analyses were carried out to identify some chemical compounds produced by the host-related substrates. Choice and no-choice experiments demonstrated that female parasitoids were poorly attracted to intact kernels with respect to the infested substrates. Furthermore, *T. elegans* females performed longer residence time on infested wheat, and they generally displayed a short-term like fidelity for this cue, remaining on it during the whole observation. Infested wheat emitted one chemical (fenchone), which is possibly linked to host larvae presence, while the feces produced by host larvae emitted some other characteristic compounds. Results demonstrated that the presence of host larvae is a key factor for *T. elegans* host habitat location, considering that the attractiveness of the undamaged wheat is irrelevant. Furthermore, parasitoid females tended to stay on attractive cues, limiting the risk of contamination of other commodities. Biological control tools may be useful to improve the efficiency of pest management programs, but their application should be carefully evaluated.

## 1. Introduction

The damage caused by insect pests during post-harvest significantly contributes to global food loss (almost 1.3 billion tons of food every year across the world) [1], influencing also food quality [2]. Yearly, over 2 billion tons of grain are harvested and stored for food and feed, but around 30% of these stored products are damaged by insect and mite pests despite the massive use of synthetic pesticides [3]. Moreover, in the last two decades, resistance to specific chemicals has been recorded for more than 504 pest species, including many stored product insect pests [3,4], reducing the effectiveness of pesticide treatments. Thus, the development of alternative approaches for pest control is a key issue to address the increasing market demand for pesticide and insect-free food, as well as to face the shortage of food commodities in many countries worldwide [5,6,7].

Among alternative approaches, biological control using natural enemies has been neglected due to legal impairments and contamination concerns. For these reasons, in many countries, the release of parasitoids and/or predators inside warehouses is not carried out, despite being legally allowed in the United States [8] and some countries of Central Europe [9]. Food regulations usually set certain levels of insect fragments in food that cannot be exceeded regardless of their origin (i.e., pest insects or natural enemies) detected by filth tests [3]. In this scenario, the intentional release of insects inside food factories discourages the storehouses because of the elevated risk of contaminants. Furthermore, to date, commercial suppliers producing natural enemies for stored product pests are limited, and there is a lack of practical expertise from pest control operators. Further research on biological control agents for stored product protection is needed to investigate their safety and efficacy, as well as to increase the knowledge about their biology and the mechanisms involved in pest/parasitoid interaction, which can be useful for their mass rearing.

Parasitoids attacking stored product pests mainly belong to the hymenopteran families Pteromalidae, Ichneumonidae, Braconidae and Bethylidae [9,10,11]. *Theocolax elegans* (Westwood) (Hymenoptera: Pteromalidae) is a cosmopolitan solitary ectoparasitoid of several stored product pests, including the coleopteran species *Rhyzopertha dominica* F. (Coleoptera: Bostrychidae), *Stegobium paniceum* L. (Coleoptera: Anobiidae), *Callosobruchus* spp. (Coleoptera: Chrysomelidae), *Sitophilus* spp. (Coleoptera: Curculionidae), and the grain moth *Sitotroga cerealella* Olivier (Lepidoptera: Gelechiidae) [12]. As a solitary parasitoid, only one *T. elegans* larva can survive at the expense of a single host larva [13]. This parasitoid species preferably parasitizes 3rd and 4th instar larvae of cereal primary feeders (i.e., pests able to damage intact kernels, whose larvae develop inside the caryopses), because *T. elegans* females do not oviposit on wandering larvae [14,15,16]. This parasitoid could reduce the population of *Sitophilus zeamais* Motschulsky, *Sitophilus oryzae* L. and *R. dominica* from 50 to 99% [13,17,18,19], while could not suppress populations of *Prostephanus truncatus* (Horn) (Coleoptera: Bostrychidae), a species closely related to *R. dominica* [20]. In contrast to other parasitoid species, *T. elegans* shows high effectiveness at medium-low temperature; the optimal developing temperature is around 25 °C and its fecundity is significantly reduced at 32 °C [21]. This characteristic is crucial for the use of this parasitoid species under field conditions. Furthermore, the synergy of *T. elegans* and *Anisopteromalus calandrae* Howard (Hymenoptera: Pteromalidae) to control *Sitophilus* spp. was investigated, and the results highlighted that *A. calandrae* was generally more effective, while *T. elegans* performed better at lower temperatures [17,18]. 

Host location is a key phase for parasitoids, and parasitoids of stored product pests can rely on a limited number of stimuli to locate their hosts and host habitats. Visual cues play a restricted role because visual stimuli are quite limited in warehouses and industries. Host-finding is mainly mediated by olfactory cues at long range and probably by vibrational and olfactory cues at short range [22]. Some studies aimed to investigate the role of cereal volatiles and host volatiles on the behaviour of *T. elegans* [23,24,25], however, it is still unclear which molecules may be involved in host selection and detection. In this study, the olfactory cues involved in the tri-trophic interaction durum wheat/*R. dominica*/*T. elegans* were investigated in choice and no-choice experiments. Furthermore, the chemical characterization of the Volatile Organic Compounds (VOCs) sampled from the host substrates was provided. 

## 2. Materials and Methods

### 2.1. Plant Material and Insect Colonies

Pesticide-free durum wheat (cv. Antalis) for insect rearing and Gas Chromatography-Mass Spectrometry (GC-MS) analyses was provided by Azienda Agricola Bognanno, Villarosa, EN, Italy (GPS coordinates: 37°35′47.0″ N, 14°10′09.8″ E). In order to ensure the absence of previous infestations, the grain was directly collected from the field on the day of harvesting; the grains were sieved using a vibrating sieve to eliminate impurities and then refrigerated at −20 °C for 48 h to avoid any possible future infestation. Samples of the collected grains were additionally subjected to different methods of analysis (floating, visual observation, and incubation) that confirmed the total absence of pests. The wheat was stored at the Department of Agriculture of the University *Mediterranea* of Reggio Calabria inside a climatic chamber at 10 °C until the beginning of the experiments.

Insect colonies were reared under controlled conditions (28 ± 1 °C, 50 ± 5% R.H.) at the Department of Agriculture of the University *Mediterranea* of Reggio Calabria. The parasitoid *T. elegans* and its host *R. dominica* were originally collected from a local milling industry (Melito Porto Salvo, Italy; GPS coordinates: 37°55′27.8″ N, 15°45′35.5″ E). 

For parasitoid rearing, 300 unsexed *Sitophilus zeamais* Motschulsky (Coleoptera: Curcolionidae) adults were placed inside a glass jar (1 L) with 500 g of rice (var. Ribe), to attain oviposition. After 1 week the grain was sieved to remove the adults and incubated for 5 weeks to ensure the development of *S. zeamais* larvae. Newly emerged *T. elegans* adults were transferred on the *S. zeamais*-infested rice. Newly emerged parasitoids were daily collected and promptly moved to fresh rearing media or used for the trials. Parasitoids were reared on a different host/plant complex to ensure that females had no previous contact with wheat grain or *R. dominica* and, therefore, could be considered naive with respect to the tested substrates [26].

To obtain the media for volatile analyses, 300 *R. dominica* adults were placed inside a glass jar (1 L) with 500 g of wheat, to attain oviposition. After 1 week the grain was sieved to remove the insects and incubated for 5 weeks to ensure the development of *R. dominica* larvae. The obtained substrate was used for VOCs analysis from infested wheat and larval feces.

### 2.2. General Methods for Bioassays

Parasitoid insects used for the behavioral assays were collected within one hour from emergence and sexed under a stereomicroscope. Female and male parasitoids (approximately 100 individuals; sex ratio 1:1) were then released inside a clean glass jar, provided with moistened cotton wicks, to ensure mating. After 48 h, *T. elegans* females (2 days old) were singly placed inside glass vials closed with a moistened cotton cap and tested within 1–2 h. 

All the behavioral assays were carried out at 25 ± 1 °C and 50 ± 5% R.H. During the bioassays, different substrates were offered to the parasitoid females in an arena made from a glass Petri dish (5.5 cm diam.) with a glass lid [27]. Parasitoid females were allowed to enter the arena by a central hole in the lid. The bioassay arena was placed inside a white chamber illuminated by artificial LED light, to exclude any possible visual cues influencing insect orientation. The observation for the single parasitoid lasted 5 min from the release [28]. After every observation, the substrates used as attractive stimuli were renewed and after 5 observations the arena was replaced by a clean one and the position of the cues was rotated. Thirty active-searching parasitoids were used for every trial. Females which did not show any searching activity (i.e., walking and drumming) after 4 min from the release were annotated (NS = no-searching), but discarded from further analyses. 

### 2.3. No-Choice and Choice Experiments

Four different stimuli were used for the bioassays: (i) undamaged intact wheat kernels (U); (ii) infested wheat kernels (I), containing 5-week-old *R. dominica* larvae and their feces; (iii) undamaged intact wheat kernels mixed with *R. dominica* larval feces (U+F); (iv) *R. dominica* larval feces (F). The kernels were previously checked for larval presence/absence under a stereomicroscope. For the trials, 500 mg of wheat kernels (around 10 kernels) were used for each stimulus. When the larval feces were included alone or mixed to wheat kernels, to reproduce the regular *R. dominica* larval excretion, 50 mg of material was used [29].

In no-choice experiments, a single stimulus was placed on one side of the arena. In choice bioassays, 2 different stimuli were placed on the opposite sides of the testing arena. The following comparisons were tested in choice trials:

1. Undamaged vs. Infested kernels
2. Undamaged vs. Undamaged kernels + Feces
3. Infested kernels vs. Undamaged kernels + Feces
4. Infested kernels vs. Feces

During the observations, the residence times (i.e., actively performing searching behavior [30]) spent by the choosing (C) females in close proximity (5 mm) or on the given substrates were recorded. Females actively searching which did not spend time in close proximity to the food sources were also recorded and labelled as no-choosing (NC) insects. The insect should spend at least 15 consecutive sec searching on the stimulus for the residence time to be recorded. Furthermore, for choice tests also the first choice (i.e., the first stimulus approached by the *T. elegans* female) was recorded.

### 2.4. Identification of Volatile Organic Compounds (VOCs)

VOCs emitted by undamaged and infested wheat and by larval feces were sampled in HS-SPME (Head Space- Solid Phase Micro-Extraction) technique by a polydimethylsiloxane (PDMS, 100 μm) Supelco^®^ fiber (Bellefonte, PA, USA). Wheat kernels (5 g) and feces (200 mg) were incubated at 27 ± 1 °C inside airtight glass vials (20 mL and 3 mL, respectively). SPME sampling was performed using the same new fiber, preconditioned according to the manufacturer’s instructions. After the incubation period, the fiber was inserted and exposed to headspace for 1 h and then desorbed for 5 min in the GC-MS injector. For every substrate, 4 replicates were provided. The sampling and desorption conditions were identical for all the samples and blanks were run before the first SPME extraction and were also randomly repeated during the injection sequences. To evaluate quantitative differences, peak areas of the same identified chemical among the different wheat samples were compared.

GC-MS analyses were performed with a Thermo Fisher TRACE 1300 gas chromatograph equipped with a MEGA-5 capillary column (30 m × 0.25 mm; coating thickness = 0.25 μm, with 10 m of pre-column) and a Thermo Fisher ISQ LT ion trap mass detector (emission current: 10 microamps; count threshold: 1 count; multiplier offset: 0 volts; scan time:1.00 s; prescan ionization time: 100 microseconds; scan mass range: 30–300 m/z; ionization mode: EI). The analytical conditions used were injector and transfer line temperature at 250 and 240 °C, respectively; oven temperature programmed from 60 to 240 °C at 3 °C min^−1^; carrier gas, helium at 1 mL min^−1^; splitless injection. The identification of VOCs was made comparing the mass spectra, as well as the linear retention indices (LRI), calculated using a series of n-hydrocarbons (C7–C30 saturated alkanes standard mixture, Supelco^®^, Bellefonte, PA, USA) [31]), and the retention times (RT) with those of commercial libraries (NIST 98 and ADAMS) and those of a library made from pure substances components of known oils and MS literature data [32,33,34,35,36].

### 2.5. Data Analysis

Binary data were analyzed using Chi-square test with one degree of freedom, while relative proportions of NC females among no-choice experiments were analyzed using Chi-square test for contingency table (3 × 2). Residence times were tested for ANOVA assumptions, normality (Shapiro–Wilk test) and homoscedasticity (Levene’s test); since these assumptions were not met also after data transformation, the data from no-choice trials were compared using the non-parametric Kruskal–Wallis H test followed by Dunn’s post hoc test for multiple comparison, while residence times from each comparison in choice bioassays were analyzed using Mann–Whitney U test. 

Relative peak areas from every VOC identified in undamaged and infested wheat kernels were Log-transformed, tested for normality (Shapiro–Wilk test) and homoscedasticity (Levene’s test) and analyzed with one-way ANOVA using “infestation” as fixed factor.

## 3. Results

### 3.1. Parasitoid Preferences for Different Host-Substrates

In no-choice trials, parasitoid females were attracted to infested wheat with respect to all other tested stimuli (Table 1). A significantly higher number of females actively host-searched in close proximity to this substrate, while very few *T. elegans* females contacted undamaged kernels (χ^2^ = 30.18; df = 3; *p* < 0.0001). Furthermore, the tested cues evoked significantly different residence times on *T. elegans* females (H_3,58_ = 9.72; *p* = 0.021), which spent significantly more time on infested wheat kernels than undamaged ones or feces, while no statistical differences were found towards residence times on undamaged grains supplemented with larval feces.

**Figure 1:**
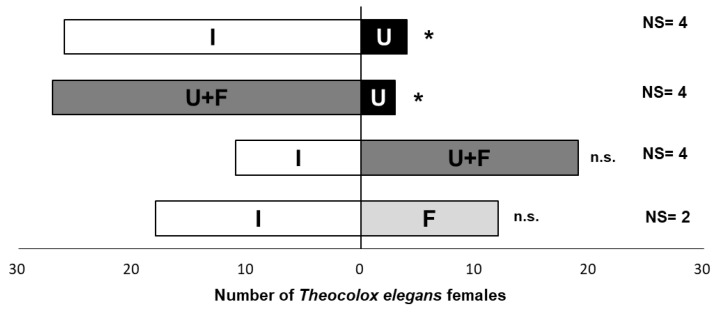
First choice of *T. elegans* females in choice experiments between paired substrates: I = *R. dominica*-Infested wheat; U = Undamaged wheat; U + F = Undamaged wheat + *R. dominica* larval feces; F = *R. dominica* larval feces. Asterisks indicate statistical differences (χ^2^ test, *p* < 0.05); n.s. = not significant. NS = no-searching female parassitoids.

The behavioral responses of parasitoid females demonstrated that *T. elegans* possessed an innate attraction for some substrates, because they started searching first on the most attractive stimuli (Figure 1). Considering the first choice (i.e., the first stimulus approached by the *T. elegans* female), undamaged wheat was significantly less attractive for parasitoid females than grains infested by *R. dominica* (χ^2^ = 16.13; df = 2; *p* = 0.0003) or undamaged kernels supplemented with host feces (χ^2^ = 19.20; df = 2; *p <* 0.0001). Furthermore, undamaged wheat added with larval feces had the same immediate attractiveness as infested wheat (χ^2^ = 2.13; df = 2; *p* = 0.34), suggesting the crucial role of feces on the host habitat location process. This hypothesis was also supported by the results of experiments with infested kernels vs. feces. *T. elegans* showed no differences in first choices for these two host substrates (χ^2^ = 1.20; df = 2; *p* = 0.55). 

In choice experiments, no significant differences were observed among the residence times when considering all the choosing insects (Total values in Table 2). However, there were differences in the number of insects actively searching on the cues; fewer female parasitoids searched on the intact wheat in comparison with the infested one (χ^2^ = 14.23; df = 2; *p =* 0.0008) or with the kernels supplemented with larval feces (χ^2^ = 21.13; df = 2; *p <* 0.0001). Conversely, the number of *T. elegans* females spending time on infested wheat was not significant different from those on intact wheat supplemented with feces (χ^2^ = 1.45; df = 2; *p =* 0.484) or on larval feces alone (χ^2^ = 2.00; df = 2; *p =* 0.367).

Evaluating the females’ selection in choice experiments according to their first choice, generally, parasitoids spent significantly more time on the substrate that was first contacted (Table 2). *T. elegans* females, first oriented to infested kernels, spent significant more time actively searching on this cue than on all the other stimuli (undamaged grain: U_1,26_ = 4.21; *p* = 0.040; undamaged grain supplemented with feces: U_1,16_ = 3.97; *p* = 0.046; feces alone: U_1,16_ = 5.14; *p =* 0.023). Similarly, females choosing first undamaged grain supplemented with feces remained on this stimulus more than on the less attractive undamaged kernels (U_1,26_ = 3.79; *p* = 0.045). Longer residence times were also displayed on feces alone with respect to infested wheat, when the females were attracted by the former (U_1,12_ = 4.33; *p* = 0.042). Considerably, *T. elegans* females did not show any short-term fidelity for intact wheat; females choosing first intact wheat did not remain on this cue during the whole duration of trials, but they spent the same time also actively searching on the other cue provided in the arena (Table 2). 

The recorded residence times suggest that the parasitoid females generally stayed on innately attractive substrates and avoided searching for other suitable host patches. This kind of short-term fidelity is supported by the number of parasitoids actively searching on the cues in choice experiments. Females choosing first infested kernels tended to stay on this substrate when contrasted with undamaged grains (χ^2^ = 20.57; df = 2; *p* < 0.0001) or only feces (χ^2^ = 18.00; df = 2; *p =* 0.0001). Conversely, there were no significant difference between the number of searching parasitoids on infested and undamaged + feces grains in choice trials, suggesting that these two substrates were equivalent (First choice = Infested: χ^2^ = 0.89; df = 2; *p =* 0.64; Undamaged + Feces: χ^2^ = 5.54; df = 2; *p =* 0.06). Notably, substrate fidelity was noted also for feces alone in comparison with infested wheat (χ^2^ = 7.14; df = 2; *p =* 0.03). Lastly, *T. elegans* females attracted to intact kernels tended to explore also the other given cue offered in the arena (intact vs. infested wheat: χ^2^ = 0.67; df = 2; *p =* 0.71; intact vs. undamaged + feces wheat: χ^2^ = 0.20; df = 2; *p =* 0.90).

### 3.2. VOCs Identification from Host-Substrates

Fourteen volatile compounds emitted by the various tested substrates were identified by GC-MS analysis following the method by Giunti et al. [37] (Table 3). Specifically, 10 volatile compounds were detected from undamaged wheat samples, while 12 VOCs infested wheat ones. The prominent volatile compounds for both grains were the alkane *n*-decane and the alcohol 1-tetradecanol. Overall, the majority of the identified VOCs were hydrocarbons, alcohols and aldehydes. 

The emissions of undamaged and infested wheat significantly differed with the emissions of four molecules. Only one compound was exclusively found in undamaged wheat, the aldehyde dodecanal. In contrast, three VOCs were emitted only by the infested kernels: the oxygenated monoterpene fenchone, the ester methyl-decanoate and the alkene 1-pentadecene. On the other hand, larval feces produced a small number of volatiles (eight compounds), all of which were also present in the volatile profile of the infested wheat. Among the three VOCs found in infested wheat, methyl-decanoate and 1-pentadecene were also detected in feces’ profiles. 

## 4. Discussion

*Theocolax elegans* females can locate potential host habitats using olfactory cues directly produced by their hosts, as well as emitted by pest-damaged substrates. The emission of HIPVs attractive for parasitoid insects has been widely investigated for crop plants [38,39], whereas the ability of grains to produce kairomones has been underestimated so far. Nevertheless, the occurrence of characteristic molecules linked to pest infestation has been demonstrated for the pteromalid parasitoid *Lariophagus distinguendus* Förster (Hymenoptera: Pteromalidae) [40,41,42,43], a generalist solitary ectoparasitoid of immature stages of at least 11 stored product pests. Concerning *T. elegans,* grain volatiles were found to be perceived and attractive for both sexes [22,23]. The ability of *T. elegans* females to discriminate between infested and intact kernels has been firstly investigated by Tang [24,25], who highlighted that host-related substances (i.e., larval saliva and frass) can play a role in parasitoid preferences. Furthermore, experienced *T. elegance* females could discriminate between *S. zeamais*-infested rice kernels and artificially damaged ones, whereas naïve females could not [24,25]. These studies provided no information about the VOC emitted by the tested rice substrates. However, the presence of specific molecules emitted by rice kernels in response to pest infestation has been investigated by recent research on the ecological interactions among three stored product pests (i.e., *S. zeamais, Tribolium confusum* du Val (Coleoptera: Tenebrionidae) and *Cryptolestes ferrugineus* (Stephens) (Coleoptera: Laemophloeidae)) [44]. 

The present study investigated the innate attractiveness of *R. dominica*-infested wheat and *R. dominca* larval feces toward *T. elegans*. Infested wheat grains can elicit longer residence times in choice and no-choice experiments, also evoking a kind of fidelity (i.e., insects remain on the first contacted substrate during the whole observation) for this substrate. Female parasitoids tend to remain on the infested wheat rather than to move and explore the other host substrates provided in the trials. This result enlightens the strong attractiveness of infested wheat and the key role of the host presence to elicit arrestment and standing on the cue. Volatile profiles from wheat infested by *R. dominica* larvae and intact wheat demonstrated that the emissions of four compounds were altered by host infestation; one chemical was produced in a higher amount by undamaged kernels, while three VOCs were associated to host larvae presence. However, only the oxygenated monoterpene fenchone was emitted by infested wheat but was absent in the volatile profile from larval feces. The absence of these molecules in *R. dominica* frass suggests that this molecule is directly emitted by infested grain. The bioactivity of fenchone has been proven also for the pteromalid parasitoid *Roptrocerus xylophagorum* (Ratzeburg) (Hymenoptera: Pteromalidae); this compound was associated with host presence (*Dendroctonus frontalis* Zimmermann and *Ips grandicollis* Eichhoff (Coleoptera: Scolytidae)) and could attract both males and females [45].

Larval feces enhance host habitat location by *T. elegans* females, increasing the arrestment duration also on undamaged grains. Olfactory cues from host feces can be exploited by several parasitoid species during their host-searching activity [46,47,48,49,50]. As an example, feces produced by several stored product pests can be attractive for *L. distinguendus* [29,41,51]. Fecal VOCs included some volatiles present also in undamaged wheat, suggesting that larval diet can influence the composition of the fecal excretions. However, the compounds methyl decanoate and 1-pentadecene were found only in infested grain profiles and feces, thus strictly correlating these compounds to host activity. 1-pentadecene is a known component of the fecal odor of *T. confusum*, which can elicit strong EAG responses and attraction toward *T. confusum* males and females [52], as well as toward the ectoparasitoid *Holepyris sylvanidis* (Brèthes) (Hymenoptera, Bethylidae) [53]. In contrast, methyl decanoate has never been detected before in the volatile profile of *R. dominica* feces, although this substrate is generally rich in linear and ramified hydrocarbons [29]. Steidle et al. [29] found a considerable amount of dominicalure 1 and 2, the species-specific male-released aggregation pheromones of *R. dominica*, in the *R. dominica* larval frass; conversely, these compounds were not detected in the present study. However, according to the authors, the conspicuous presence of adult pheromones in larval feces was due to the rearing conditions, because adult beetles were never completely removed from the larval cultures used for the experiments. 

From an applied point of view, results on the limited attraction of undamaged wheat grain were encouraging because the presence of hosts is a key factor for host habitat location. Biological control for stored product pests is quite limited, either for operative criticisms, but also because of reluctance and concerns about the introduction of organisms in food thereby potentially increasing insect residues. Nevertheless, Flinn et al. [13,21] demonstrated that the augmentative release of *T. elegans* in wheat bins to control *R. dominica* infestation led to a significant reduction of insect fragments at the end of the storage period. The low number of *T. elegans* females approaching intact kernels suggests that parasitoids would concentrate on host infested materials and would avoid unsuitable substrates, lowering the risk of contamination of other products or raw materials. 

The release of *T. elegans* can be used in combination with other biorational tools, in order to decrease the application of synthetic pesticides and to improve the whole efficiency of integrated pest management (IPM) programs in the food industry. Indeed, fumigation with carbon dioxide (about 60%, for 22 days) of bag stacks, containing rye and wheat, could effectively suppress the *R. dominica* adult population, while *T. elegans* parasitoids could survive as pupae, granting control of the survived host larvae [54]. Biological control agents, thus, can be combined with other low-impact approaches to improve the efficacy, as well as the sustainability, of control programs for stored product pests damaging food during post-harvest storage. Furthermore, the identification of host-related semiochemicals could be useful to improve the monitoring of parasitoid population density in field conditions [55] and may be used to increase the efficacy of parasitisation rates [38,42]. 

Further research is required to evaluate through electroantennographic assays whether the identified compounds can be perceived by parasitoids, the bioactivity of these VOCs and the impact of females’ experience on host habitat location. More information about the role of visual, tactile and vibrational stimuli on host and host habitat location would be necessary to better understand *T. elegans* behavior. Therefore, deeper knowledge about chemically mediated host/parasitoid interactions is essential to design appropriate and effective IPM programs, which can include biological agents to increase the eco-friendly methods to control stored product pests. 

## 5. Conclusions

Overall, this research investigated for the first time the host habitat location behavior of *T. elegans* females on the complex *R. dominica*-durum wheat, highlighting the key role of host feces for parasitoid host habitat location. Results from choice and no-choice trials proved that cues from infested grain are more attractive for naïve female parasitoids with respect to stimuli from undamaged kernels. Furthermore, the infested host substrate elicited a sort of fidelity in *T. elegans* females attracted to *R. dominica*-infested wheat. Conversely, fidelity was not reported for undamaged wheat, suggesting that parasitoids did not spend much time searching for host larvae on unsuitable substrates.

## Figures and Tables

**Table 1:** Resident times (Mean ± SE) of *T. elegans* females in no-choice trials. Capital letters indicate significant differences among the numbers of choosing females (χ^2^ test, *p* < 0.05). Letters indicate significant differences among the times spent on the substrates (Kruskal–Wallis H test; *p* < 0.05).

Substrates	NS ^1^ Females (N ^4^)	NC ^2^ Females (N)	C ^3^ Females (N)	Residence Time (s)
Infested	3	4	26 A	129.0 ± 15.0 a
Undamaged + Feces	3	14	16 B	97.8 ± 20.0 ab
Feces	2	12	18 B	73.9 ± 11.7 b
Undamaged	5	25	5 C	40.6 ± 12.1 b

^1^ No-searching; ^2^ No-choosing; ^3^ Choosing; ^4^ Number.

**Table 2:** Residence times (Mean ± SE) of *T. elegans* females in choice trials with paired cues. Asterisks (*) indicate significant differences between the number of insects spending Table 2 test; *p* < 0.05) or between the residence times spent on the cues (Mann–Whitney U test; *p* < 0.05) within the same row.

Trial	Cue A	Cue B	First Choice	Cue A (N ^1^)	Cue B (N)		Residence Time Cue A (s)	Residence Time Cue B (s)	
1	Infested	Undamaged	Infested	26	2	*	170.0 ± 20.6	16.5 ± 1.5	*
			Undamaged	2	4	ns ^2^	158.0 ± 26.0	143.3 ± 51.3	ns
			**Total**	**28**	**6**	*	**169.1 ± 19.1**	**101.0 ± 42.0**	**ns**
2	Undamaged	Undamaged + Feces	Undamaged	3	2	ns	121.0 ± 75.0	103.5 ± 67.5	ns
			Undamaged + Feces	0	27	*	0.0 ± 0.0	162.7 ± 19.0	*
			**Total**	**3**	**29**	*	**121.0 ± 75.0**	**158.7 ± 18.2**	**ns**
3	Infested	Undamaged + Feces	Infested	11	7	ns	163.8 ± 27.7	74.9 ± 16.3	*
			Undamaged + Feces	7	19	ns	147.3 ± 15	170.5 ± 21.9	ns
			**Total**	**18**	**26**	**ns**	**157.4 ± 17.6**	**144.7 ± 18.5**	**ns**
4	Infested	Feces	Infested	18	0	*	179.4 ± 22.1	0.0 ± 0.0	*
			Feces	2	12	*	73.0 ± 9.0	213.8 ± 21.9	*
			**Total**	**20**	**12**	**ns**	**168.8 ± 21.2**	**213.8 ± 21.9**	**ns**

^1^ Number. ^2^ not significant.

**Table 3:** Volatile Organic Compunds (VOCs) identified by GC-MS analyses from different host substrates: undamaged and *R. dominica*-infested wheat, and *R. dominica* larval feces. Relative percentages of peak areas are mean ± standard error of three replicates. Asterisks (*) indicate significant differences between relative percentages of the same compound between undamaged and infested wheat (One-way ANOVA; *p* < 0.05).

Compound	LRI ^1^ Calculated	LRI Literature	Undamaged (%)	Infested (%)	F ^2^	*p ^3^*	Feces (%)
Decane	1000	1000	33.58 ± 8.21	11.84 ± 4.35	0.6	0.47	34.67 ± 9.63
Limonene	1038	1037	tr ^4^	3.19 ± 3.14	0.61	0.46	-
Fenchone	1090	1087	-	4.78 ± 2.83	8.99	0.02 *	-
Nonanal	1101	1102	-	3.6 ± 2.98	2.99	0.13	tr
1-Decanol	1274	1274	1.19 ± 1.03	3.32 ± 1.09	3.92	0.1	5.88 ± 1.36
Tridecane	1299	1300	2.2 ± 1.32	-	3.00	0.13	-
Undecanal	1310	1306	0.95 ± 0.66	1.63 ± 0.69	0.48	0.51	tr
Methyl-decanoate	1331	1326	-	3.43 ± 1.3	7066.36	<0.001 *	8.48 ± 2.29
Tetradecane	1398	1400	tr	-	1.00	0.36	tr
Dodecanal	1412	1409	5.9 ± 3.56	-	7.99	0.03 *	-
1-Pentadecene	1491	1491	-	1.67 ± 0.6	8.98	0.02 *	0.45 ± 0.25
1-Hexadecene	1585	1589	8.85 ± 8.65	0.38 ± 0.38	0.001	0.98	-
Tetradecanal	1614	1611	6.07 ± 2.69	tr	1.11	0.33	-
1-Tetradecanol	1681	1676	36.67 ± 17.96	46.78 ± 10.61	1.53	0.26	47 ± 14.34
**Total**			**97.04 ± 2.55**	**98.23 ± 1.69**			**96.78 ± 3.03**

^1^ Linear Retention Index; ^2^ F value of one-way ANOVA; ^3^ Probability of one-way ANOVA; ^4^ traces.

## Data Availability

The data that support the findings of this study are available from the corresponding authors [G.G. and O.C.] upon reasonable request.

## References

[1] Mhlanga N., Seidler E., Njie D., Gallat S., Lamb J., Morgan N., Zorya S., Diaz Rios L. (2010). FAO/World Bank Workshop on Reducing Post-Harvest Losses in Grain Supply Chains in Africa.

[2] Nayak M.K., Daglish G.J., Athanassiou C.G., Arthur F.H. (2018). Importance of Stored Product Insects. Recent Advances in Stored Product Protection.

[3] Boyer S., Zhang H., Lempérière G. (2012). A review of control methods and resistance mechanisms in stored-product insects. Bull. Entomol. Res..

[4] Sparks T.C., Nauen R. (2015). IRAC: Mode of action classification and insecticide resistance management. Pestic. Biochem. Physiol..

[5] Campolo O., Giunti G., Russo A., Palmeri V., Zappalà L. (2018). Essential Oils in Stored Product Insect Pest Control. J. Food Qual..

[6] Mbata G., Warsi S. (2019). *Habrobracon hebetor* and *Pteromalus cerealellae* as Tools in Post-Harvest Integrated Pest Management. Insects.

[7] Campolo O., Verdone M., Laudani F., Malacrinò A., Chiera E., Palmeri V. (2013). Response of four stored products insects to a structural heat treatment in a flour mill. J. Stored Prod. Res..

[8] Environtal Protection Agency (EPA) (1992). Parasitic and predaceous insects used to control insect pests; exemption from a tolerance. Fed. Reg..

[9] Schöller M., Prozell S., Suma P., Russo A., Athanassiou C.G., Arthur F.H. (2018). Biological Control of Stored-Product Insects. Recent Advances in Stored Product Protection.

[10] Schöller M., Flinn P.W., Subramanyam B., Hagstrum D.W. (2000). Parasitoids and predators. Alternatives to Pesticides in Stored-Product IPM..

[11] Amante M., Schöller M., Suma P., Russo A. (2017). Bethylids attacking stored-product pests: An overview. Entomol. Exp. Appl..

[12] Schöller M., Prozell S., Al-Kirshi A.G., Reichmuth C. (1997). Towards biological control as a major component of integrated pest management in stored product protection. J. Stored Prod. Res..

[13] Flinn P.W., Hagstrum D.W., Mcgaughey W.H. (1996). Suppression of beetles in stored wheat by augmentative releases of parasitic wasps. Environ. Entomol..

[14] Toews M.D., Phillips T.W., Cuperus G.W. (2001). Effects of wheat cultivar and temperature on suppression of *Rhyzopertha dominica* (Coleoptera: Bostrichidae) by the parasitoid *Theocolax elegans* (Hymenoptera: Pteromalidae). Biol. Control.

[15] Van den Assem J., Kuenen D.J. (1958). Host finding of *Choetospila elegans* Westw. (Hym. Chalcid.) a parasite of *Sitophilus granarius* L. (Coleopt. Curcul.). Entomol. Exp. Appl..

[16] Dlamini B.E., Amornsak W. (2014). Effect of host age on progeny production of *Theocolax elegans* (Westwood) (Hymenoptera: Pteromalidae) reared on *Sitophilus zeamais* (Motschulsky) (Coleoptera: Curculionidae). Kasetsart J. Nat. Sci..

[17] Wen B., Smith L., Brower J.H. (1994). Competition between *Anisopteromalus calandrae* and *Choetospila elegans* (Hymenoptera: Pteromalidae) at different parasitoid densities on immature maize weevils (Coleoptera: Curculionidae) in corn. Environ. Entomol..

[18] Wen B., Brower J.H. (1995). Competition between *Anisopteromalus calandrae* and *Choetospila elegans* (Hymenoptera: Pteromalidae) at different parasitoid densities on immature rice weevils (Coleoptera: Curculionidae) in wheat. Biol. Control.

[19] Flinn P.W. (1998). Temperature Effects on Efficacy of *Choetospila elegans*. J. Econ. Entomol..

[20] Helbig J. (1998). Ability of naturally occurring parasitoids to suppress the introduced pest *Prostephanus truncatus* (Horn) (Coleoptera, Bostrichidae) in traditional maize stores in Togo. J. Stored Prod. Res..

[21] Flinn P.W., Hagstrum D.W. (2001). Augmentative releases of parasitoid wasps in stored wheat reduces insect fragments in flour. J. Stored Prod. Res..

[22] Germinara G.S., De Cristofaro A., Rotundo G. (2009). Antennal olfactory responses to individual cereal volatiles in *Theocolax elegans* (Westwood) (Hymenoptera: Pteromalidae). J. Stored Prod. Res..

[23] Germinara G.S., De Cristofaro A., Rotundo G. (2016). Electrophysiological and Behavioral Responses of *Theocolax elegans* (Westwood) (Hymenoptera: Pteromalidae) to Cereal Grain Volatiles. Biomed. Res. Int..

[24] Tang Q. (2016). *Sitophilus zeamais*-Induced rice grain volatiles: Attractiveness towards the generalist parasitoid wasp, *Theocolax elegans*. Pak. J. Zool..

[25] Tang Q. (2016). Olfactory responses of *Theocolax elegans* (Hymenoptera, Pteromalidae) females to volatile signals derived from host habitats. J. Hymenopt. Res..

[26] Giunti G., Benelli G., Messing R.H., Canale A. (2016). Early adult learning affects host preferences in the tephritid parasitoid *Psyttalia concolor* (Hymenoptera: Braconidae). J. Pest Sci..

[27] Steidle J.L.M., Lanka J., Muller C., Ruther J. (2001). The use of general foraging kairomones in a generalist parasitoid. Oikos.

[28] Desneux N., Barta R.J., Hoelmer K.A., Hopper K.R., Heimpel G.E. (2009). Multifaceted determinants of host specificity in an aphid parasitoid. Oecologia.

[29] Steidle J.L.M., Steppuhn A., Ruther J. (2003). Specific Foraging Kairomones Used by a Generalist Parasitoid. J. Chem. Ecol..

[30] Benelli G., Revadi S., Carpita A., Giunti G., Raspi A., Anfora G., Canale A. (2013). Behavioral and electrophysiological responses of the parasitic wasp *Psyttalia concolor* (Szépligeti) (Hymenoptera: Braconidae) to *Ceratitis capitata*-induced fruit volatiles. Biol. Control..

[31] Van Den Dool H., Kratz P.D. (1963). A generalization of the retention index system including linear temperature programmed gas-liquid partition chromatography. J. Chromatogr..

[32] Adams R.P. (1995). Identification of Essential Oil Components by Gas Chromatography/Mass Spectrometry.

[33] Davies N.W. (1990). Gas chromatographic retention indices of monoterpenes and sesquiterpenes on methyl silicon and Carbowax 20M phases. J. Chromatogr. A.

[34] Jennings W. (1980). Qualitative Analysis of Flavor and Fragrance Volatiles by Glass Capillary Gas Chromatography.

[35] Masada Y. (1976). Analysis of Essential Oils by Gas Chromatography and Mass Spectrometry.

[36] Stenhagen E., Abrahamsson S., McLafferty F.W. (1974). Registry of Mass Spectral Data.

[37] Giunti G., Campolo O., Laudani F., Algeri G.M., Palmeri V. (2020). Olive fruit volatiles route intraspecific interactions and chemotaxis in *Bactrocera oleae* (Rossi) (Diptera: Tephritidae) females. Sci. Rep..

[38] Godfray H.C.J. (1994). Parasitoids: Behavioral and Evolutionary Ecology.

[39] Dicke M., Baldwin I.T. (2010). The evolutionary context for herbivore-induced plant volatiles: Beyond the “cry for help”. Trends Plant Sci..

[40] Steidle J.L.M., Fischer A.A., Gantert C. (2005). Do grains whisper for help? Evidence for herbivore-induced synomones in wheat grains. Entomol. Exp. Appl..

[41] Benelli G., Pacini N., Conti B., Canale A. (2013). Following a scented beetle: Larval faeces as a key olfactory cue in host location of *Stegobium paniceum* (Coleoptera: Anobiidae) by *Lariophagus distinguendus* (Hymenoptera: Pteromalidae). Chemoecology.

[42] Steiner S., Steidle J.L.M., Ruther J. (2007). Host-associated kairomones used for habitat orientation in the parasitoid *Lariophagus distinguendus* (Hymenoptera: Pteromalidae). J. Stored Prod. Res..

[43] Steidle J.L.M., Steppuhn A., Reinhard J. (2001). Volatile cues from different host complexes used for host location by the generalist parasitoid *Lariophagus distinguendus* (Hymenoptera: Pteromalidae). Basic Appl. Ecol..

[44] Giunti G., Palmeri V., Algeri G.M., Campolo O. (2018). VOC emissions influence intra- and interspecific interactions among stored-product Coleoptera in paddy rice. Sci. Rep..

[45] Pettersson E.M., Sullivan B.T., Anderson P., Berisford C.W., Birgersson G. (2000). Odor perception in the bark beetle parasitoid *Roptrocerus xylophagorum* exposed to host associated volatiles. J. Chem. Ecol..

[46] Takabayashi J., Takahashi S. (1989). Effects of host fecal pellet and synthetic kairomone on host-searching and postoviposition behavior of *Apanteles kariyai*, a parasitoid of *Pseudaletia separata*. Entomol. Exp. Appl..

[47] Turlings T.C.J., Tumlinson J.H., Heath R.R., Proveaux A.T., Doolittle R.E. (1991). Isolation and identification of allelochemicals that attract the larval parasitoid, *Cotesia marginiventris* (Cresson), to the microhabitat of one of its hosts. J. Chem. Ecol..

[48] Agelopoulos N.G., Dicke M., Posthumus M.A. (1995). Role of volatile inforchemicals emitted by feces of larvae in host-searching behavior of parasitoid *Cotesia rubecula* (Hymenoptera: Braconidae): A behavioral and chemical study. J. Chem. Ecol..

[49] Alborn H.T., Lewis W.J., Tumlinson J.H. (1995). Host-specific recognition kairomone for the parasitoid *Microplitis croceipes* (Cresson). J. Chem. Ecol..

[50] Chiu-Alvarado P., Rojas J.C. (2010). Behavioural responses of bethylid parasitoid species of the coffee Berry borer to chemicals cues from host and non-host dust/frass. BioControl.

[51] Steidle J.L.M., Fischer A. (2000). Quantity does matter: How feces are used for host stage selection by granary weevil parasitoid *Lariophagus distinguendus*. J. Chem. Ecol..

[52] Verheggen F., Ryne C., Olsson P.O.C., Arnaud L., Lognay G., Högberg H.E., Persson D., Haubruge E., Löfstedt C. (2007). Electrophysiological and behavioral activity of secondary metabolites in the confused flour beetle, *Tribolium confusum*. J. Chem. Ecol..

[53] Fürstenau B., Adler C., Schulz H., Hilker M. (2016). Host Habitat Volatiles Enhance the Olfactory Response of the Larval Parasitoid *Holepyris sylvanidis* to Specifically Host-Associated Cues. Chem. Senses.

[54] Banks H.J., Sharp A.K. (1979). Insect control with CO_2_ in a small stack of bagged grain in a plastic film enclosure. Aust. J. Exp. Agric..

[55] Robacker D.C., Weaver K.M., Hendry L.B. (1976). Sexual communication and associative learning in the parasitic wasp *Itoplectis conquisitor* (Say). J. Chem. Ecol..

